# Supplementary material for: The interactome of intact mitochondria by cross-linking mass spectrometry provides evidence for coexisting respiratory supercomplexes
Source: Mol Cell Proteomics. 2017 Dec 8;17(2):216–32. doi: 10.1074/mcp.RA117.000470 (PMC5795388; doi:10.1074/mcp.RA117.000470)
Supplement: Supplemental Data [file supp_RA117.000470_133922_0_supp_23978_fzffwf.zip › spectra_annotation/mito_DR_spectra_annotation/306-1-4-1-5-1.pdf]

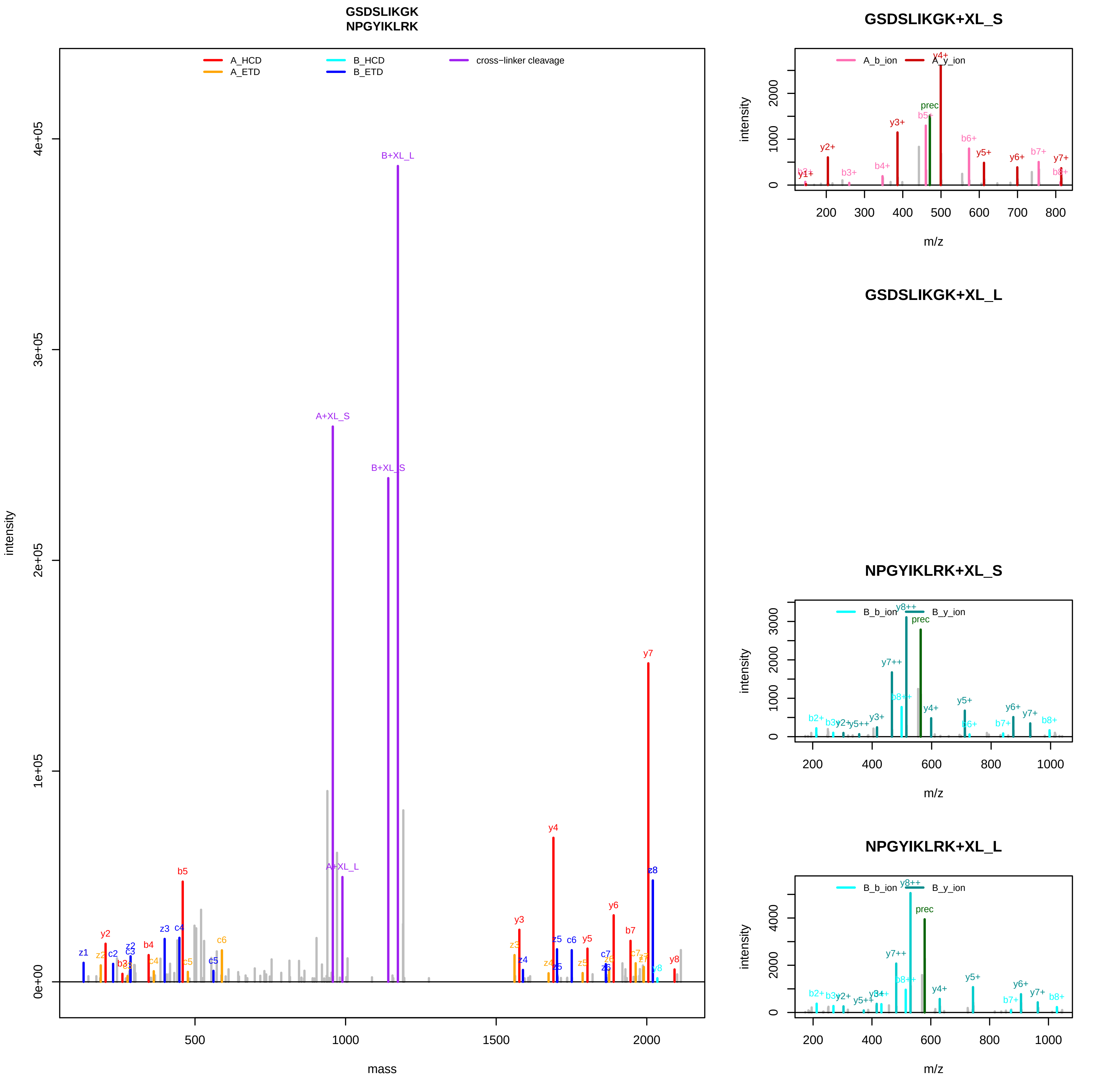

GSDSLIKGK
NPGYIKLRK
A_HCD
A_ETD
B_HCD
B_ETD
cross−linker cleavage
B+XL_L
A+XL_S
B+XL_S
A+XL_L
y7
y4
y3
y5
y6
b7
y8
z8
b5
b4
b3
y2
z1
z2
c2
c3
z3
c4
c5
c6
c7
z4
z5
z6
z7
c4
z5
c6
intensity
mass
4e+05
3e+05
2e+05
1e+05
0e+00
500
1000
1500
2000
GSDSLIKGK+XL_S
A_b_ion
A_y_ion
prec
y1+
y2+
y3+
y4+
y5+
y6+
y7+
b2+
b3+
b4+
b5+
b6+
b7+
b8+
m/z
GSDSLIKGK+XL_L
NPGYIKLRK+XL_S
B_b_ion
B_y_ion
y8++
y7++
b8++
y5++
y3+
y4+
y5+
y6+
y7+
b2+
b3+
y2+
b6+
b7+
b8+
NPGYIKLRK+XL_L
